# The roles of photo-carrier doping and driving wavelength in high harmonic generation from a semiconductor

**DOI:** 10.1038/s41467-017-01899-1

**Published:** 2017-11-22

**Authors:** Zhou Wang, Hyunwook Park, Yu Hang Lai, Junliang Xu, Cosmin I. Blaga, Fengyuan Yang, Pierre Agostini, Louis F. DiMauro

**Affiliations:** Department of Physics, The Ohio State University, Columbus, OH 43210 USA

## Abstract

High-harmonic generation from gases produces attosecond bursts and enables high-harmonic spectroscopy to explore electron dynamics in atoms and molecules. Recently, high-harmonic generation from solids has been reported, resulting in novel phenomena and unique control of the emission, absent in gas-phase media. Here we investigate high harmonics from semiconductors with controllable induced photo-carrier densities, as well as the driving wavelengths. We demonstrate that the dominant generation mechanism can be identified by monitoring the variation of the harmonic spectra with the carrier density. Moreover, the harmonic spectral dependence on the driving wavelength is reported and a different dependence from the well-known one in gas-phase media is observed. Our study provides distinct control of the harmonic process from semiconductors, sheds light on the underlying mechanism and helps optimize the harmonic properties for future solid-state attosecond light sources.

## Abstract (web summary)

The properties of high harmonic generation from solids are not fully understood. Here, Wang et al. control the photo-carriers injected in a semiconductor to distinguish interband and intraband contributions to different high harmonics, and investigate the wavelength dependence of the harmonics.

## Introduction

The attosecond electronic response of semiconductors to intense femtosecond laser fields is a new frontier in ultrafast science. The sub-laser-cycle phenomena in semiconductors, from petahertz transient absorption^1,2^ to optical-field-induced current^3^, provide potential access to ultrafast optoelectronics for signal processing^4^. High-order harmonic generation (HHG) from semiconductors driven by intense fields has been demonstrated as a novel window into the electronic structure and dynamics^5–10^. It has been shown that the HHG process conveys information for reconstructing the electronic band structure^7,11^, and provides a possible alternative route for generating attosecond pulses^10^.

**Fig. 1:**
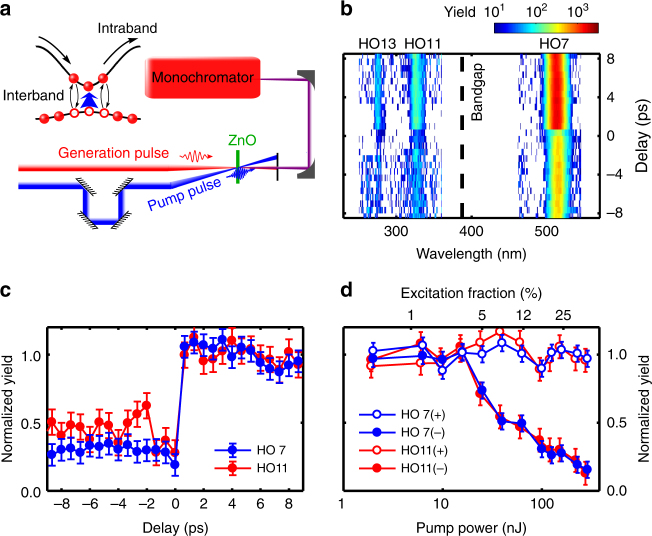
Experimental measurements of HHG from photoexcited ZnO. **a** Experimental arrangement: a 0.4 µm pump pulse generates abundant carriers in the conduction and valence band (blue arrow). The temporally delayed generation pulse centered at 3.5 µm drives the high-harmonic generation (HHG), which is collected by a monochromator. **b** Harmonic spectra from the mid-infrared pulse as a function of delay between the pump and generation pulses. 7th, 11th and 13th order harmonics are marked as HO7, HO11 and HO13, while the bandgap is marked by the black dashed line. When the crystal is photoexcited beforehand, the harmonic yield is reduced, as shown at negative delays. The 9th harmonic around 390 nm is saturated by the pump beam scattered into the monochromator and is removed. **c** Integrated yield of the 7th (below bandgap) and 11th (above bandgap) order harmonics as a function of delay. The error bars in yield are given by the fluctuation of HHG signals. The error bars in delay are negligible. **d** Effects of pump strength on HHG. For positive delays (labeled as (+)), the HHG yield is not affected by the later-arrived pump pulse, while for negative delays (labeled as (−)), the HHG yield is reduced by pre-excitation. This suppression effect does not depend on the relative polarization of the pump pulse (Supplementary Fig. 1). The excitation percentage is estimated based on the pump photon fluence and the absorption efficiency

In contrast to HHG from gas-phase atoms which is well described by the semi-classical model^12,13^, the interaction of an intense, ultrafast pulse with the periodic structure of a semiconductor crystal results in two distinct mechanisms for HHG (Fig. 1a), i.e., the interband and intraband transitions^14–16^. For the interband process, the electron–hole pair is generated, accelerated by the laser field and subsequently recombines coherently, emitting a harmonic photon. Conversely, in the intraband picture, electrons (holes) photoexcited into the conduction (valence) band are driven by the laser field and oscillate in the Brillouin zone, known as the Bloch oscillations. The corresponding anharmonic electronic current in the bands results in harmonic emission.

The balance between mechanisms can change in different materials or spectral regimes. Vampa et al. conducted a two-color phase measurement in ZnO which concluded that the interband transition was more significant than the intraband one^8^, at variance with the earlier report^5^. Furthermore, they showed that the phases could be used to reconstruct the band structure^11^. Alternatively, using a terahertz driver, Schubert et al.^6^ demonstrated sub-cycle control of HHG in gallium selenide and observed dynamical Bloch oscillations. A more recent study in SiO_2_ employing a streaking method^10^ in the extreme ultraviolet regime verified the intraband model^7^.

In addition, novel controls have been demonstrated in terms of the crystal-orientation-dependent harmonic polarization^17,18^, ellipticity dependence^19^ and emission phases^18^, which is absent in the gas-phase HHG. Nanostructures have also been utilized to alter the harmonics^20,21^. In this paper, we show that manipulating the carrier density via seeded photoexcitation provides a different form of control and aids in clarifying the underlying mechanisms of solid HHG. Furthermore, the driving laser wavelength, a conventional tuning variable of the gas-phase HHG, can also be applied to explore and manipulate the solid HHG.

In this article, we investigate the HHG dependence on the controlled carrier density doped by photoexcitation and explore the effect of the driving wavelength. By studying the degree of incoherent photoexcited carriers, we identify the mechanism for both below-bandgap and above-bandgap HHG. HHG from different driving wavelengths provides an alternative approach to investigate and control the HHG from semiconductors. Comparing the experiment to a two-band Bloch model we show that the HHG mechanisms differentially evolve with the driving wavelength within ZnO. The spectral wavelength dependence shows a distinct difference to the gas-phase HHG. Our study provides a further step toward engineering the material characteristics and controlling laser wavelengths for optimizing HHG properties.

## Results

### Photo-carrier doping

When generating harmonics from a single pulse, the carriers (i.e., electrons and holes) are coherently populated and driven by the same laser field. To study the effect of carrier density, our experiment uses a pump pulse (photon energy *ħω* ≥ *E*_g_) to dope excessive incoherent carriers in the semiconductor. A delayed intense mid-infrared (MIR) pulse generates high harmonics from the doped crystal. Note that the dephasing time of the coherent electron–hole pair in semiconductors typically ranges from a few to tens of femtoseconds^22^. In previous studies, high-order sidebands are generated when the pump and generation pulses are temporally overlapped, i.e. before the electron–hole pair dephases^23,24^. In our study, the generation pulse arrives much later than the dephasing time and alone drives coherent HHG. The HHG is produced in a 200-µm-thick ZnO (0001) single crystal driven by a ∼1 TW cm^−2^ MIR pulse centered at a wavelength of 3.5 µm. Figure 1a illustrates the experimental arrangement. A non-collinear pump pulse centered at 0.4 µm in wavelength photoexcites the ZnO crystal across the direct bandgap (3.2 eV). The delayed MIR generation pulse can drive the intraband transitions of the photoexcited carriers in one band and/or coherently induce excitation and recombination between bands.

Figure 1b shows the HHG spectra as a function of delay between the 0.4 µm pump and the 3.5 µm generation pulses. The below-bandgap (7th order) and above-bandgap (11th and 13th order) harmonics of the MIR can be identified on the spectrogram (*E*_g_ is marked by the black dashed line). At positive delays, the pump pulse arrives after the generation pulse and thus has no effect on HHG, whereas for negative delays, it arrives before the generation pulse and the harmonics are generated from the photoexcited crystal. In contrast to the unexcited crystal at positive delays, the HHG yield for all orders reduces with pre-excitation. Figure 1c plots the integrated yield of the 7th and 11th order harmonics and clearly shows that populating the conduction/valence band with electrons/holes inhibits the HHG both below and above the bandgap. The suppression is time-independent since the spontaneous decay time for the excited carriers is hundreds of picoseconds^25^, much longer than the delay. In addition, we verify that the transmission of the MIR pulse is unaffected by the pump pulse (Methods section), thus ruling out the contributions from macroscopic effects such as the refractive index variation.

In order to investigate the role of the carrier density, the HHG yield is measured as a function of pump energy for two delays, ±5 ps, as shown in Fig. 1d. The variation of the pump energy alters the population of photoexcited carriers (<1–25%, 10^20^–10^21^ cm^−3^). Again positive delays result in no change in the HHG yield. However, for negative delays the yield is strongly suppressed for both harmonic orders and depends on the pump energy. Thus a higher carrier density does not facilitate the HHG but only reduces it.

**Fig. 2:**
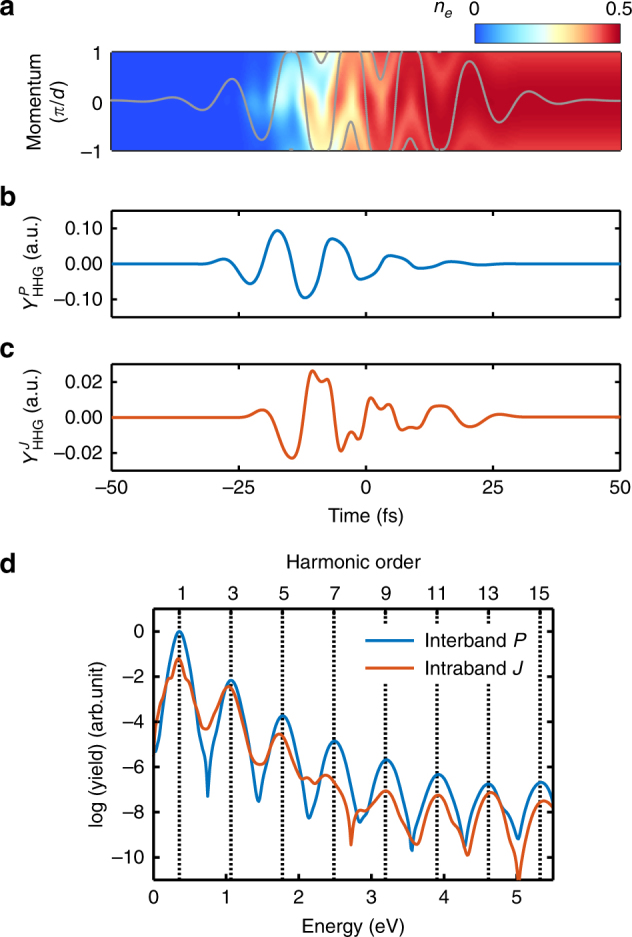
Simulation of HHG from a single mid-infrared pulse. **a** The evolution of electron population in the conduction band. The population oscillates in momentum space, corresponding to the intraband transition. The gray line shows the classical motion of a conduction electron driven by the laser field. The alternative increase and decrease of the population in a sub-laser-cycle period along the gray line is given by the interband transition. **b** Time-dependent harmonic yield $Y_{{\mathrm{HHG}}}^P\left( t \right)$ in atomic units (a.u.) from the interband contribution (polarization). **c** Time-dependent harmonic yield $Y_{{\mathrm{HHG}}}^J\left( t \right)$ from the intraband contribution (current). **d** High-harmonic energy spectra from the interband (blue) and intraband (red) transitions by Fourier-transforming (**b**, **c**), respectively

To interpret the experimental observations, a two-band, one-dimensional model based on semiconductor Bloch equations is applied to calculate the time-dependent interband polarization term and the intraband current term^14^. Figure 2a shows the evolution of the electron population in the momentum space of the conduction band driven by a MIR generation pulse alone. The signatures for both the intraband and interband transitions are identifiable. First, the electron population $n_e\left( t \right)$ excited into the conduction band oscillates in the momentum space, constituting the Bloch oscillations associated with the intraband transitions. Second, the population along the oscillating trace increases and decreases alternatively within every laser cycle, which indicates the population variation caused by the interband transitions. The time-dependent harmonic yields $Y_{{\mathrm{HHG}}}^{P/J}(t)$ from the interband and intraband transitions are calculated and are separately plotted in Fig. 2b, c, respectively. Figure 2d shows the HHG spectra obtained by a Fourier transform of $Y_{{\mathrm{HHG}}}^{P/J}(t)$. The model suggests that the harmonic yield from the interband term dominates over the intraband contribution for a single generation pulse.

**Fig. 3:**
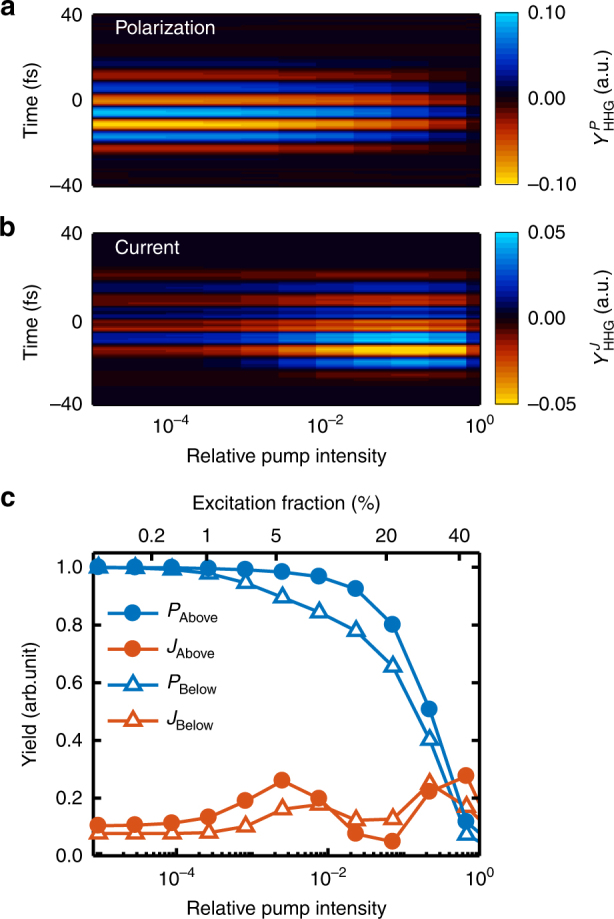
Calculated HHG from photoexcited ZnO at different pump strengths. The relative pump intensity has units of a fraction of the generation intensity of 1.2 TW cm^−2^. **a** Time-dependent harmonic yield $Y_{{\mathrm{HHG}}}^P\left( t \right)$ from the interband transition as a function of pump intensity. No dependence is observed at low pump intensities, and the yield decreases rapidly at high pump intensities at a range above 10^−1^. **b** Time-dependent harmonic yield $Y_{{\mathrm{HHG}}}^J\left( t \right)$ from the intraband transition as a function of pump intensity. With a weak pump at a range above 10^−^^4^, pre-photoexcited carriers enhance the yield. **c**, The energy-integrated yield as a function of pumping intensity or excitation fraction. Blue lines are from the interband polarization contribution (labeled as P) and red lines are from the intraband contribution (labeled as J). Both the above-bandgap harmonic yield (integrated over 3.3–4.2 eV, filled circle) and the below-bandgap harmonic yield (integrated over 1.5–2.0 eV, open triangle) behave similarly. The pump-dependence of $Y_{{\mathrm{HHG}}}^P$ is consistent with the measurements in Fig. 1d

In order to simulate the photoexcitation geometry, a 0.4 µm electric field is introduced before the generation field (Methods section). A certain amount of electron (hole) population is pumped into the conduction (valence) band, after which the delayed generation pulse drives HHG from the interband polarization and the intraband current. The delay plays no role as long as the two pulses do not overlap. The time-dependent responses $Y_{{\mathrm{HHG}}}^{J/P}(t)$ from the interband polarization and the intraband current at various pump strengths are plotted in Fig. 3a, b, respectively. Figure 3a shows that the time-dependent response $Y_{{\mathrm{HHG}}}^P(t)$ from the interband polarization at various pump intensities is weakly dependent on the pump at low intensities but eventually decreases rapidly at high pump intensities. This effect caused by the depletion of carriers results in fewer electrons remaining in the valence band to be excited and coherently recombined. This is analogous to the ground state depletion in the gas-phase HHG, and can be understood with a simplified two-level system (Supplementary Fig. 2). In contrast, the intraband current term plotted in Fig. 3b shows that the response $Y_{{\mathrm{HHG}}}^J(t)$, thus HHG, is enhanced with an increasing pump intensity. This is consistent with the previous observation that injected photoexcited carriers can greatly enhance the terahertz emission via the Bloch oscillations in a superlattice under a constant external field^26^. At the highest pump intensities, the HHG yield decreases since the strong excitation results in a uniform momentum space distribution of electrons (holes) occupying the conduction (valence) band, thus reducing the net current (see discussion in Supplementary Fig. 3).

Figure 3c summarizes the calculated integrated HHG yield for harmonic energies below and above the bandgap. Besides the obvious dominance of the interband polarization term, the two terms show a distinct difference with the pump intensity. While the pre-excitation enhances the intraband contribution, the interband contribution is only suppressed, thus our measurement in Fig. 1d can identify these contributions. We observe for above-bandgap and below-bandgap HHG only a constant yield with an increasing carrier density followed by a suppression, consistent with the behavior of the interband mechanism. Our approach can access the mechanism for individual harmonics either below or above the bandgap, and our observation suggests a dominant contribution to the below-bandgap harmonics from interband emission, which is at variance with some previous theories^15,27^. This is not surprising since virtual states within the bandgap can be non-resonantly excited in a manner similar to the below-threshold harmonic emission from gas-phase atoms.

### Driving wavelength dependence

Our MIR experiment supports the interpretation that HHG emission in ZnO is an interband process between the conduction and valence bands, while the intraband contribution remains relatively weak. However, our calculations suggest that this situation will change at longer wavelengths.

**Fig. 4:**
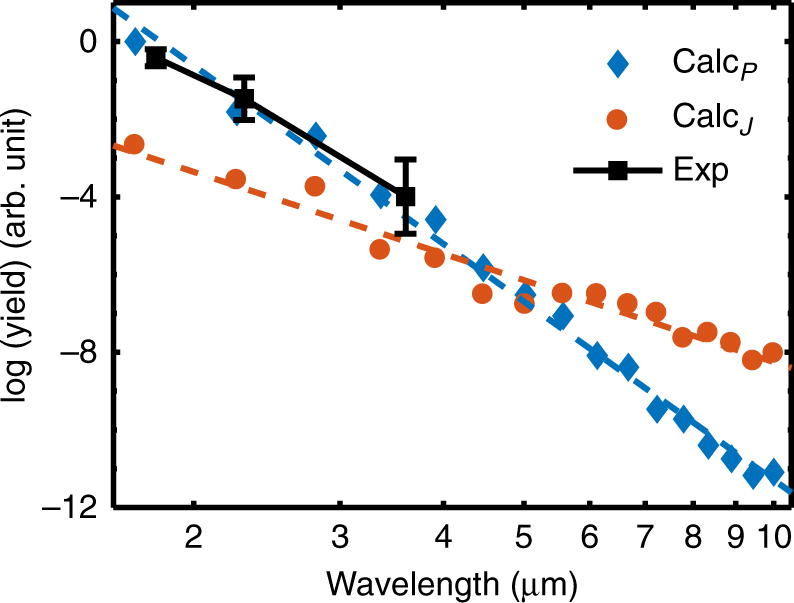
Wavelength dependence of HHG yield. The calculated yield (integrated over 2.0–4.0 eV) from either the interband transition (blue diamonds) or the intraband transition (red dots) decreases monotonically. A fitted slope of *λ* ^−(15.3 ± 0.8)^ from the interband polarization is steeper than the fitted slope of *λ* ^−(7.0 ± 0.9)^ from the intraband current. At longer wavelengths, the contribution from the intraband transition exceeds that from the interband transition (Supplementary Fig. 5). Our experimental measurement (black squares) (Supplementary Fig. 6) covers the mid-infrared range where the interband contribution dominates. The fitted slope of *λ* ^−(11.4 ± 2.9)^ from the experiment (black squares) matches the calculation well, and is much steeper than the well-established atomic case^28,29^. The error bars in the figure are estimated according to the variation of focal sizes and pulse durations at each wavelength

Figure 4 shows the calculated HHG yield (within a constant energy range) as a function of driving wavelength from unexcited ZnO and our measured values (black squares). The calculation shows that the yield from both the interband and intraband transitions decreases with increasing wavelengths. Our measured HHG yield from 2–4 μm agrees surprisingly well with the calculated wavelength dependence of the interband curve, where intraband contribution is negligible. Furthermore, the decrease in the ZnO HHG efficiency scales with a *λ* ^−(11.4 ± 2.9)^ wavelength dependence, which is much stronger than the *λ* ^−(5-6)^ atomic case^28,29^. However, most importantly, the interband contribution declines faster than the intraband contribution. Physically, a longer driving wavelength requires more photons to drive the interband transition resulting in a decreased HHG yield. In addition, the short dephasing time can also suppress the emission at longer wavelengths^15^. Alternatively, the wavelength-dependent contribution from the intraband transition depends on both the charge density and its momentum space distribution. Thus the wavelength dependence of the two contributions is expected to diverge.

Our results are at variance with an earlier calculation which showed an increased absolute yield from the intraband transition at longer wavelengths^15^. Furthermore, our calculations suggest that at longer wavelengths, e.g., in the far-infrared or terahertz regime, the overall absolute HHG yield will decrease but the predominant emission will switch to the intraband process. However in this regime, an increase of carrier density should be an effective method for enhancing the HHG emission. This can stimulate future work, for example, doping the crystal to enhance harmonic yield while maintaining the dense harmonic comb for applications such as high-harmonic spectroscopy.

**Fig. 5:**
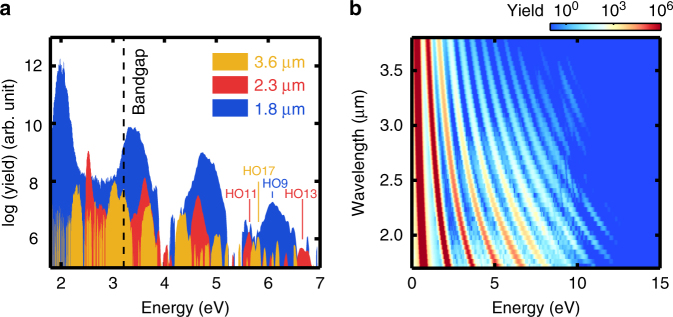
Cutoff comparison at different driving wavelengths from unexcited ZnO. **a** Measured spectra at different wavelengths. The intensities are slightly lower than Fig. 4 to observe the cutoffs. Spectra beyond 7 eV is not detected due to instrumental limitations. The cutoff from 1.8 µm appears not shorter than the cutoffs from long wavelengths at 2.3 and 3.6 µm. **b** The calculated cutoff appears not sensitive to the driving wavelength, in contrast to the well-known atomic case

Regarding the cutoff, different scalings with driving wavelength have been predicted theoretically so far. Some studies claimed a linear wavelength dependence^5,27,30,31^, while others found it to be wavelength independent^16,32,33^. Here we remark first that, in contrast to atoms/molecules, the HHG spectrum from semiconductors decreases gradually with the order without an obvious cutoff. Defining a cutoff as the highest frequency detected, previous works found that it increased linearly with the field strength^5–7^. With the same definition of cutoff, we observed that in our wavelength measurement, the cutoff at 1.8 μm is not shorter than that at 3.6 μm in Fig. 5a. At 3.6 µm, cutoff at H17 at 5.9 eV can be resolved while H19 at around 6.5 eV is not discernible from background noise. In comparison, at 2.3 µm, H13 still appears at around 6.9 eV although barely distinguishable from the background, which indicates a similar cutoff to 3.6 µm. At 1.8 µm, the 9^th^ harmonics still has significant yield around a similar energy range, and it does not show a shorter cutoff in comparison to 3.6 and 2.3 µm.

Such behavior is distinct from the atomic cases where the cutoff scales with the square of the wavelength, but in agreement with a previous theoretical study^28^ which shows almost no dependence on wavelengths. This is also verified by our calculation in Fig. 5b, where the cutoffs are almost the same across the wavelength range. The cutoff energy at a short driving wavelength appears even slightly higher, which is probably due to a more perturbative nature when the driving photon energy is relatively large compared to the bandgap. Note that the cutoff can be higher than the maximum bandgap energy, since the strong field can distort the band structure and modulate the bandgap.

## Discussion

In conclusion, by controlling the injected photo-carriers in a time-resolved pump-probe experiment and comparing to a time-dependent semiconductor Bloch model, we can identify the contributions from the interband and intraband transitions for different harmonics. We show that interband transitions dominate both below- and above-bandgap HHG emission from ZnO crystals driven by intense MIR pulses. A strong wavelength dependence of the measured HHG yield agrees with our model in the regime dominated by the interband transitions. We expect that as the wavelength increases, the emission will switch to the intraband transition. The novel wavelength dependence of the cutoff is measured as well. Our study can hopefully not only provide an alternative method to investigate the HHG mechanism in semiconductors, but also inspire future work on engineering the semiconductor and optimizing driving pulses for better HHG performance.

## Methods

### Experimental setup

In the experiment, MIR (3–4 µm) light is generated by a home-built optical parametric amplifier (OPA) pumped by a commercial 1 kHz 12 mJ Ti:sapphire amplifier with 80 fs pulse duration (Spitfire Ace, Spectra-Physics). The depleted pump of the OPA is frequency-doubled to produce a 0.4 µm pump pulse. A concave mirror with 100 mm focal length focuses the pump and MIR generation pulses in a non-collinear geometry, so that the pump pulse can be blocked from propagating into the monochromator. The generation pulse propagates along the optical axis of the 200-µm-thick ZnO (0001) sample (MTI, Corp.). The HHG from the generation pulse is refocused into a monochromator and collected by a gated ICCD camera (PI-MAX, Princeton Instrument). For the 0.4 µm pump alone, no harmonic signal is observed, which will be vacuum ultraviolet if existing.

The delay between the pump pulse and the generation pulse is scanned by a motorized translational stage. A wide delay range up to 10 ps is studied, and no temporal dependence is observed when the pump pulse arrives early (or later) than the generation pulse without overlap. In the study of the pump power, the generation pulse arrives about 5 ps before or after the pump pulse. In this case, the input energy of the pump pulse is controlled by a neutral density (ND) filter wheel. In general, excitation of more than about 10% of valence electrons can result in lattice instability and disorder^34^. However, in our experiment no irreversible changes are observed from pumping the crystal.

To exclude the effect from a photoexcitation-induced variation in the refractive index, the transmission of the generation pulse is carefully monitored with delay and found to be reduced by <4% for the strongest excitation, thus this would have a minor effect on the harmonic yield.

For studying the wavelength dependence, additional MIR light (1.7–2.3 µm) is generated by a commercial superfluorescence-based OPA (HE-TOPAS, Light Conversion). In this study, both 1.7–2.3 µm OPA and 3–4 µm OPA are pumped by a home-built 1 kHz Ti:sapphire system with a pulse duration of 100 fs. The plateau height at different wavelengths is compared, and calibrated based on the focal size for a comparison of HHG efficiency. The beam sizes at 1.8 and 2.3 µm are measured by a CCD camera at focus, while that at 3.6 µm is calculated based on the beam size measured by an infrared camera (Electrophysics, Corp.) before the focusing optics. The intensities are estimated based on the input power and the beam size. The error bars in Fig. 4 provide the uncertainty of the measured yield ratio: 25% variation of focal sizes and 20% variation of pulse durations. In order to investigate the cutoff, the intensities at all three wavelengths are reduced by half, since the instrumental setup limits the detection range below about 7 eV.

Note that for above-bandgap harmonics, the absorption length is around or below 100 nm^35^, thus the detected HHG comes from a thin layer on the back side of the crystal.

### Modeling

A quantum description of the electronic dynamics under external fields is applied based on the semiconductor Bloch equations^6,9,14^. The equations include two bands of ZnO^36^:

1 $$i\hbar \frac{\partial }{{\partial t}}p_k = \left( {\varepsilon _k^c - \varepsilon _k^v - i\frac{\hbar }{{T_2}}} \right)p_k - \left( {1 - n_k^e - n_k^h} \right)d_kE\left( t \right) + ieE(t)\frac{\partial }{{\partial k}}p_k$$

2 $$\hbar \frac{\partial }{{\partial t}}n_k^{e(h)} = - 2{\mathrm{Im}}\left[ {d_kE\left( t \right)p_k^{\mathrm{*}}} \right] + eE\left( t \right)\frac{\partial }{{\partial k}}n_k^{e(h)}$$

$p_k$ is the polarization and $n_k^{e(h)}$ is the electron (hole) population in the conduction (valence) band at crystal momentum *k*. $T_2$ is the dephasing time of an electr on–hole pair, which is around 5 fs^21^. A discussion on the dephasing time can be found in the Supplementary Fig. 4. $d_k$ is the dipole transition element between two bands^37^, which is adjusted to match the experimentally measured spectra in terms of harmonic order content.

Previous studies show that inclusion of multiple bands can introduce quantum interferences between bands^9,38^, as well as multiple plateau structure on harmonic spectra^16,39,40^. However, we consider only two bands here for simplicity, since the yield of the main plateau can be well described by considering only the conduction and valence band, excluding higher bands^16^. In addition, we applied the band structure along the Γ–M direction from the literature^36^.

A 30 fs MIR sine-squared pulse centered at 3.5 µm with 1.2 TW cm^−2^ drives the interband polarization and the intraband current. The pulse duration in the calculation is shorter than the experiment in order to avoid saturation before the peak of the generation pulse.

The macroscopic polarization (labeled as *P*) from the interband transition and electronic current (labeled as J) from the intraband transition are calculated based on $p_k$ and $n_k^{e(h)}$:

3 $$Y_{{\mathrm{HHG}}}^P(t) = \mathop {\sum }\limits_k \omega [d_kp_k(t) + c.c.]$$

4 $$Y_{{\mathrm{HHG}}}^J(t) = \mathop {\sum }\limits_k [\frac{1}{\hbar }\frac{{\partial \varepsilon _k^e}}{{\partial k}}en_k^e + \frac{1}{\hbar }\frac{{\partial \varepsilon _k^h}}{{\partial k}}\left( { - e} \right)n_k^h]$$

The time-dependent harmonic yield $Y_{{\mathrm{HHG}}}^{P/J}\left( t \right)$ is then Fourier-transformed into the energy domain to obtain the HHG frequency spectra.

In the photoexcited HHG calculation, a 0.4 µm pump pulse with 30 fs pulse duration is applied 100 fs before the 3.5 µm generation pulse (Supplementary Fig. 3). The pump pulse populates the conduction (valence) band with electrons (holes). The excitation percentage is extracted at the end of the pump pulse before the generation pulse arrives. The generation pulse drives the HHG from the pre-excited bands. When varying the excitation power, the Bloch equations are solved for HHG with different initial conditions effectively to study the effect of pre-excitation.

In the wavelength-dependent calculation, a pulse duration of 60 fs and intensity of 1.2 TW/cm^2^ at all wavelengths is applied for a wavelength-dependent efficiency comparison. In comparison to the photoexcitation study, the longer pulse duration used here is to avoid few-cycle effects at far-infrared regime. The calculated yield is summed over the energy range of 2–4 eV for the efficiency comparison between different driving wavelengths.

### Data availability

The data that support the findings of this study are available from the corresponding authors on request.

## Electronic supplementary material

Supplementary Information
