# Supplementary material for: The roles of photo-carrier doping and driving wavelength in high harmonic generation from a semiconductor
Source: Nat Commun. 2017 Nov 22;8:1686. doi: 10.1038/s41467-017-01899-1 (PMC5698516; doi:10.1038/s41467-017-01899-1)
Supplement: Supplementary file 1 — Supplementary Information [file 41467_2017_1899_MOESM1_ESM.pdf]

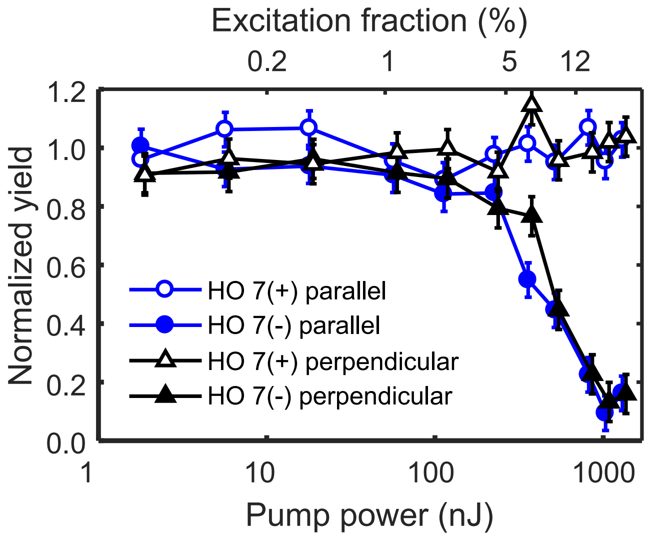

Supplementary Figure 1. Parallel versus perpendicular pump polarizations. For either pump polarization, at positive delays (open symbols), the 7th order harmonic yield is not affected by the pump pulse, while for negative delays (filled symbols), the high harmonic yield is reduced by pre-excitation regardless of the pump polarization. The difference of pump power in comparison to Fig. 1d in the main text comes from a different beam size, which is about three times larger here. The excitation percentage (top scale) is estimated based on the pump photon fluence and the absorption efficiency. The error bars are given by the fluctuation of high harmonic signals.

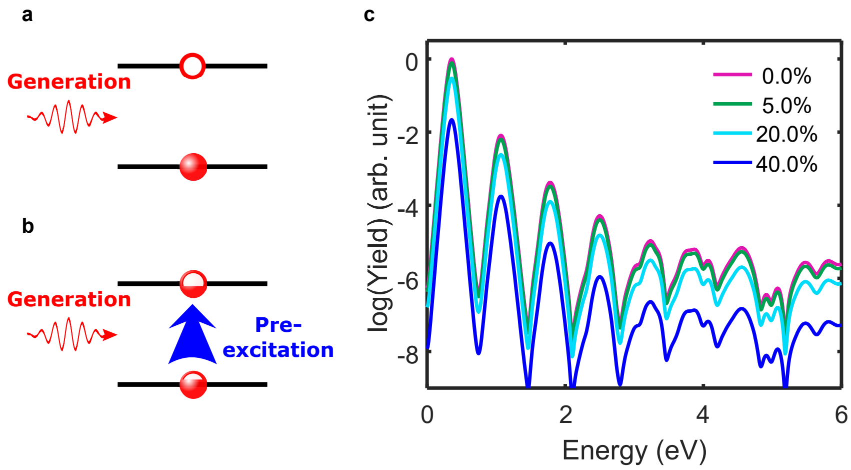

Supplementary Figure 2. Effect of pre-excitation in a two-level picture. The optical Bloch equations of the two-level system, with the same parameters discussed in the Methods, is solved for the high harmonic generation (HHG)[1,2], while no intraband transition exists here since no band structure is involved. This is similar to the case where transitions can happen only at the Γ point, where k=0[3]. **a**, Schematic diagram for HHG in a two-level system driven by the generation pulse alone. **b**, Schematic diagram for HHG in the same system but with a distinct initial condition, where the upper level is manually populated to mimic the pre-excitation before the arrival of the generation pulse. **c**, HHG spectra with different excitation percentage. A suppression of the HHG yield by the pre-excitation is observed. Although decoupling the interband transition from the intraband transition potentially leads to a modification of the HHG spectrum[4], the two-level system here sheds light on the effects of pre-excitation in the interband transition, especially considering the case here where the interband contribution dominates.

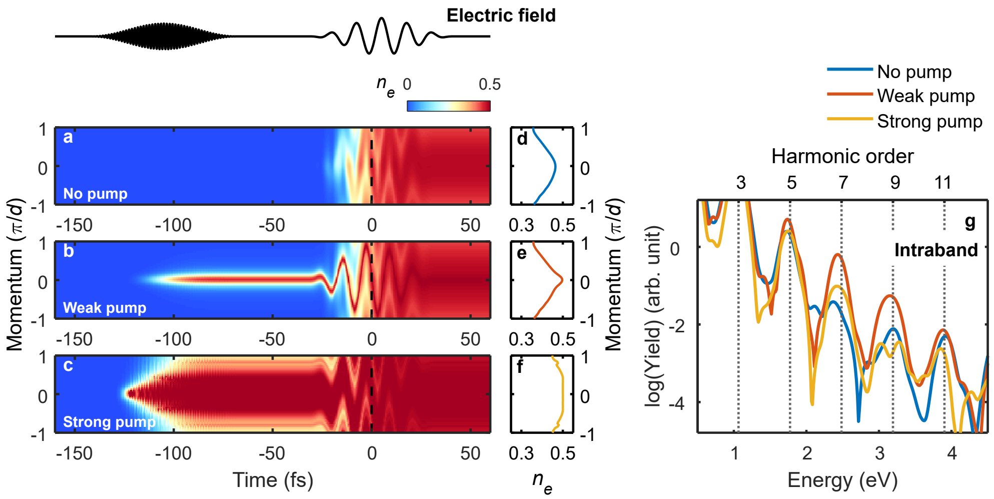

Supplementary Figure 3. Calculated effect of the pump strength on the intraband current. a-c, the conduction band population in the momentum and time space for different pump powers. The laser electric field is plotted on the top (black line) where the pump pulse (left) is followed by a mid-infrared pulse (right). **d-f**, the corresponding population distribution in the momentum space at t=0 (marked by black dashed lines in **a-c**). The case without pump (**a**, **d**) is the same as Fig. 2a in the main text. With a weak pump (**b**, **e**) (excitation percentage of 5%, at relative pump strength of 1.6×10^{-3} in Fig. 3c of the main text), abundant charge is created and localized in the momentum space, and then driven by the mid-infrared field to oscillate in the conduction band, thus enhances the intraband current in comparison to **a**. However, with a strong pump (**c**, **f**) (excitation percentage of 35%, at relative pump strength of 0.36 in Fig. 3c of the main text), although more carriers populate the band, the distribution is more uniform in **f** comparing to **e**, which reduces the net current. **g**, HHG spectra from the intraband transition (Bloch oscillations) only. The weak pump (red line) enhances the HHG yield (blue line). However, further increasing pump strength starts suppressing the 7th to 11th harmonics (yellow line). A stronger pump will further reduce the harmonics including the lower orders. Note that y-axis is in log scale, and the enhancement varies from twice to more than an order of magnitude.

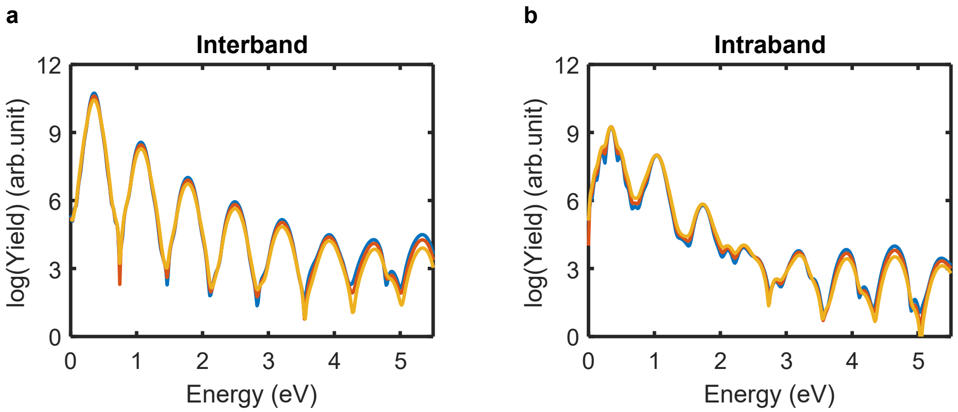

Supplementary Figure 4. Effects from the dephasing time. As the carrier density changes, due to the carrier-carrier collision, the dephasing time T_2 in principle also decreases. It was empirically fitted that $T_2 \propto N^{-0.3}$, where N is the carrier density[5,6]. Based on the estimation, the carrier density at the peak of the pulse with or without pump is not sufficiently distinct to alter the dephasing time significantly. HHG spectra under the three dephasing times of 5 fs (blue), 4 fs (red), and 3 fs (yellow) are plotted for the interband transitions (**a**) and the intraband transitions (**b**). Higher carrier densities slightly lower the HHG yield, but overall difference within this range is minor.

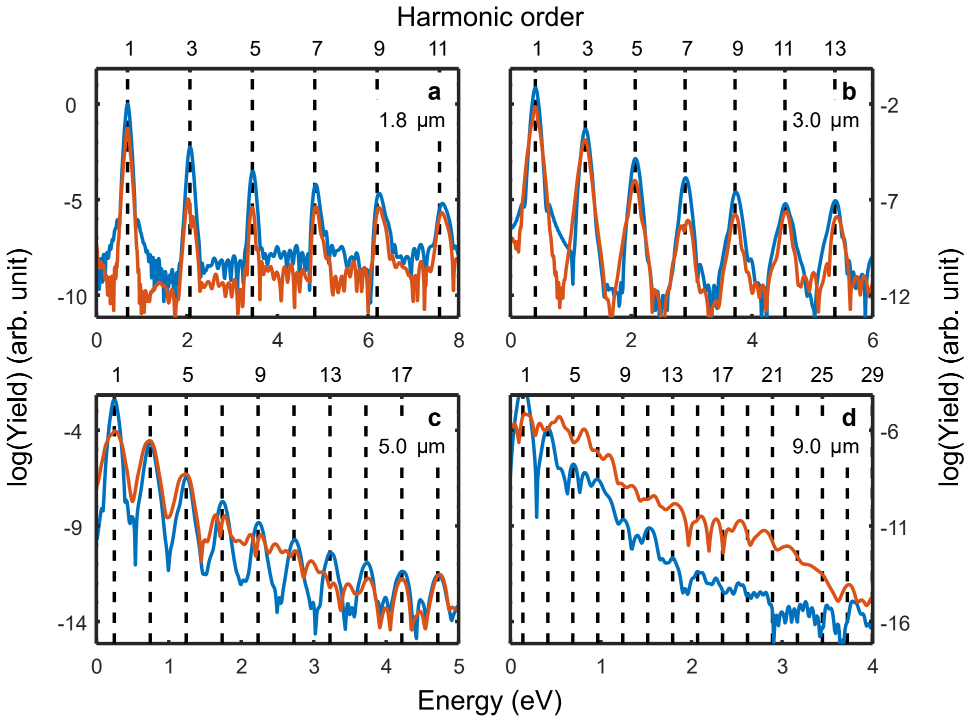

Supplementary Figure 5. Calculated high harmonic spectra from different driving wavelengths from unexcited ZnO. The calculations are performed at (a) 1.8 μm, (b) 3.0 μm, (c) 5.0 μm and (d) 9.0 μm. As the wavelength increases, the yield from both the interband transition (blue line) and the intraband transition (red line) decreases. At short wavelengths, the contribution from the interband transition dominates. As the wavelength increases, the contribution from the intraband transition takes over.

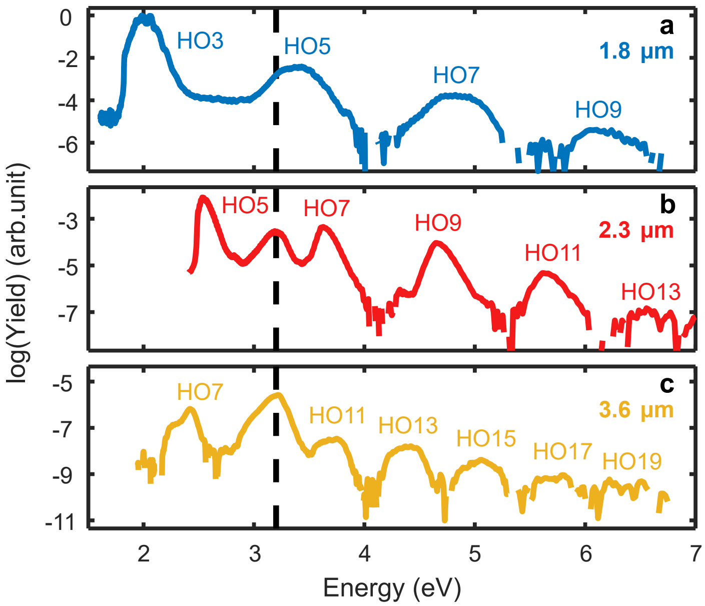

Supplementary Figure 6. Measured high harmonic spectra at different driving wavelengths from unexcited ZnO. The spectra are measured at (a) 1.8 μm, (b) 2.3 μm and (c) 3.6 μm. The experimentally measured HHG yield, scaled based on the focal conditions for a comparison of HHG efficiency, decreases dramatically as the driving wavelength increases. The black dashed line marks the position of fluorescence peak at the direct bandgap. The fluorescence signal at the shortest driving wavelength (1.8 μm) is overtaken by the harmonic signal. The HHG peak width is broadened since the monochromator slit is wide open to collect all harmonic signal, at the expense of the frequency resolution.

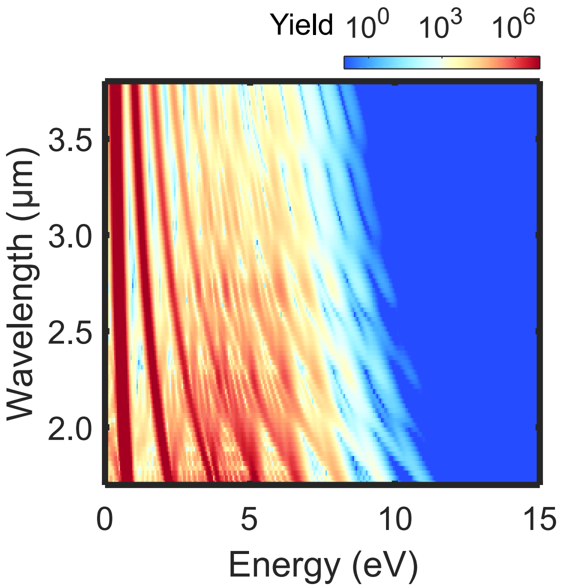

Supplementary Figure 7. Simulated wavelength-dependent high harmonic cutoff using a two-level system. The cutoffs are calculated with a two-level system in the wavelength regime where the interband transition dominates. The calculated cutoff is not very sensitive to the driving wavelength, same to the full calculation in Fig. 5 of the main text. The cutoff from the two-level system can be estimated analytically as $E_{\text{cutoff}} = \hbar\sqrt{\omega_0^2 + 4\Omega^2}$, where ω_0 is the transition frequency and Ω is the Rabi frequency[2]. It appears independent of the driving wavelength.

Supplementary References

1. Wegener, M. *Extreme Nonlinear Optics* (Springer, 2006).

2. Gauthey, F. I., Garraway, B. M. & Knight, P. L. High harmonic generation and periodic level crossings. *Phys. Rev. A* **56**, 3093 (1997).

3. Wu, M., Browne, D. A., Schafer, K. J. & Gaarde, M. B. Multilevel perspective on high-order harmonic generation in solids. *Phys. Rev. A* **94**, 063403 (2016).

4. Golde, D., Meier, T., Koch, S. W. High harmonics generated in semiconductor nanostructures by the coupled dynamics of optical inter- and intraband excitations. *Phys. Rev. B* **77**, 075330 (2008).

5. Becker, P. C. *et al.* Femtosecond photon echoes from band-to-band transitions in GaAs. *Phys. Rev. Lett.* **61**, 1647 (1988).

6. Arlt, S., Siegner, U., Kunde, J., Morier-Genoud, F. & Keller, U. Ultrafast dephasing of continuum transitions in bulk semiconductors. *Phys. Rev. B* **59**, 14860 (1999).
